# Utilization of mid-upper arm circumference as discharge tool for children in outpatient therapeutic program, Ethiopia

**DOI:** 10.1017/jns.2022.98

**Published:** 2022-11-10

**Authors:** Abera Lambebo, Dessalegn Tamiru, Tefera Belachew

**Affiliations:** 1 Department of Public Health, College of Health Science, Debre Berhan University, Debre Berhan, Ethiopia; 2 Department of Nutrition and Dietetics, Faculty of Public Health, Jimma University, Jimma, Ethiopia

**Keywords:** MUAC, SAM, Under five, Oromia, Ethiopia

## Abstract

Mid-upper arm circumference (MUAC) is simple to use and inexpensive in Ethiopia; both MUAC and target weight are employed, although the time to cure for MUAC is not indicated. The present study is aimed to determine cure time of MUAC for children in outpatient therapeutic program. A prospective cohort study was conducted among 414 severe acute malnourished under-five children admitted to selected health twenty-two posts from 1 February to 30 July 2021, in Oromia, Ethiopia. Data were coded, entered to Ep-data version 4.2 software, and transferred to SPSS for windows version 25 software for analysis. The Multivariate Cox Proportional Hazards model was used to fit independent determinants of time to cure. All tests were two-sided and statistical implications at *P*-values < 0⋅05. In the present study, the minimum week for a cure was 4 weeks, the maximum was 16 weeks and the overall time to cure severe acute malnutrition as measured by MUAC is judged to be 10 at 95 % CI (9⋅65–10⋅35). Families with six or more members are 2⋅16 times more at risk, children from homes with the lowest wealth index are at 1⋅4 times more risk, and children from food insecure families were 2⋅61 times more likely to require long-term treatment for MUAC. In the present study, the time to cure severe acute malnutrition by MUAC is determined as 10 weeks. Moreover, family size, low wealth index, and household food insecurity were risks to delay in cure time MUAC.

## Introduction

Children with severe acute malnutrition (SAM) are nine times more likely to die compared with well-nourished children. In addition, the management of severe acute malnutrition is critical for child survival and is a key cost-effective component of the scaling up nutrition outline for addressing undernutrition^(1)^.

In the resource, limited sceneries where malnutrition is common, accurate measurement of weight and height may be a challenge. Mid-upper arm circumference (MUAC) is easier to quantify and interpret and it is similar in boys and girls and is relatively constant from 6 months to 5 years which leads to avoiding the obligation to determine the exact age^(2)^. In nations with a high incidence of undernutrition, timely, correct screening at the community level is vital to identify children with wasting^(3)^. Moreover, the MUAC is used as a substitution to evaluate wasting in children. However, its legitimacy thrives in controversies^(4)^.

A greater number of drawbacks are recognised in the usage of the existing, as evidence to answer the question about the relevance of strategy to identify the children at risk of acute malnutrition. There is doubt among practitioners and difference of opinion among practitioners and academic experts, concerning the strength of the indication to support this recommendation and the penalties for children identified with SAM using weight-for-height *Z*-score or (WHZ) only^(5)^.

The MUAC and WHZ or weight-for-length *Z*-score or (WLZ) have different associations with body composition, and length influences these associations differently, and MUAC acts more like a composite index of poor growth indexing^(6)^.

Weight gain is routinely monitored to assess hydration and growth during the treatment of children with complex severe acute malnutrition. However, changes in weight and MUAC gain speeds over time are barely described^(7)^.

Another challenge for MUAC or MUAC grounded on a solitary cut-off value for all the children less than 5 years of age has been used for numerous years as another nutritional status index for children through scarcities or refugee catastrophes, and as an extra screening tool in non-emergencies. However, it has newly been interrogated whether MUAC is age and sex-autonomous. A Study in Senegal suggests that MUAC is superior to WHZ in classifying high-risk children in the community and using both WHZ less than −3 and MUAC less than 115 mm increases specificity but decreases compassion to classify high-risk children. There is no advantage for programmes in combining WHZ and MUAC to identify high-risk children^(8)^.

A problem that is encountered in admission standards for severe acute undernutrition is based on children with a weight-for-height below −3 sd, MUAC less than 110 mm, and with medical difficulties like loss of appetite and oedema but this is more meticulous more than 99 % over the age range 6–60 months and discharge only 15–20 % of weight gain^(9)^.

Moreover, another study suggests that a cut-off of <11⋅5 cm is not enough to detect SAM children so it needs to increase to ensure early diagnosis of children and to avoid miss of children from referral for management of malnutrition. Therefore, to decrease the risk and death of undernutrition-sensitive cut-off point MUAC is needed^(10)^. In addition, a weight-for-height *Z*-score ≥ −2 suggests that short children with stumpy MUAC fail to gain extreme fat during supplementation, and the practice of length as a criterion for gaging MUAC to determine treatment eligibility was outdated in policy and practice^(11)^.

The anthropometric gauge that is used to identify severe acute malnutrition should also be used to trace whether a child has grasped nutritional retrieval, i.e. if MUAC is used to identify that a child has severe acute malnutrition. Likewise, if weight-for-height is used to categorise that a child has severe acute malnutrition, then weight-for-height should be used to measure and check nutritional recapture^(12)^.

However, the World Health Organization on its 2013 severe acute management guideline suggests that there is no difference in weight-for-height and MUAC; for a programme utilising MUAC weight gain is 15–20 % afterward oedema vanishes for the programme utilising weight-for-height: weight-for-height > –11 standard deviations or weight gain is 15–20 % after oedema dissolves^(13)^.

As MUAC is easy to practice for classifying and follow up severe acute malnutrition among under-five children. However, the time to cure MUAC is not identified and the present study aimed to identify the time to cure for MUAC and factors affecting the survival of under-five children from severe acute malnutrition for MUAC in Ethiopia.

## Methods

### Study area and period

The study was conducted in Habro Woreda, West Hararghe Zone, Oromia, Ethiopia. Habro Woreda is found in the West Hararghe Zone of Oromia Regional State. The capital city of Habro Woreda is called Gelemso which is located about 326 km East of Addis Ababa and 76 km away from Chiro Zonal Town of West Hararghe. The total under-five population of the Woreda was 44, 243, 6–59 months was 40 392, and households of the Woreda were 56 099. The Woreda has 32 rural kebeles (localities) and 5 urban kebeles (localities). It also has thirty-two health posts and seven health centres and one general hospital, all of the health facilities, except the hospital are providing OTP services. The main income of the population depends on agriculture^(14)^. The study period was from 1 February to 30 July 2021.

#### Study design

Prospective cohort study.

#### Population

Under-five children were admitted for severe acute malnutrition in twenty-two health posts during the study period.

### Sample size and sampling procedures

The sample size was determined based on the expectations with a recovery rate of 93⋅3 %, a hazard ratio of 0⋅38^(15)^, a 5 % margin of error, 95 % CI and a power of 80 %. Then, it was considered by medcalc©version 119.1.1.3 survival analysis (log-rank test) at http://www.medcal.org^(16)^. Finally, by bearing in mind 10 % for failure to follow up, the total study sample size was 414.

### Sampling procedure

In the West Hararghe Zone of Oromia Regional State, Habro Woreda, there are 32 rural kebeles (localities) and 5 urban kebeles (localities) and 32 health posts providing OTP service. From them twenty-two rural health posts were randomly selected, namely, Health post, Chafe 12 HP, Bareedaa HP, Wenne kallo HP, Medda Jaalalaa HP, Booraa HP, Maqrac HP, Ifaa Gemechu HP, Busoytu HP, Melkaa balloo HP, Odaa Mudaa HP, Daraaraa HP, Kallachaa HP, Gerbi Teka HP, Ibsaa HP, Kufa kaas HP, Dikkichaa HP, Bedada HP, Haro Carcar HP, Lallisaa HP, Lagabeeraa HP, odaa Ananii HP and Gadisa HP.

### Measurement

Data were collected from the children's admitted for SAM management in health posts; a prospective cohort study was conducted for 6 months from February to July in randomly selected health posts. Data collection tools include socio-economic data (age, sex, food security and household wealth index), time (time for primary admission, time for discharge and anthropometric measurements (height, weight, MUAC, oedema). One supervisor and twenty-two data collectors were trained and involved in data collection. Data collectors received a one-day training on data collection tools and interpersonal reliability on Anthropometric measurement was checked by the technical error of measurement (TEM) after that organised to gather data once the main investigator was convinced about their competency. The primary investigator of the study and the supervisors critically followed the data collection procedure to minimise missing information and discrepancies.

### Operational definition

#### Wasting

Weight-for-height *Z*-score < −2. It often indicates recent and severe weight loss, although it can also persist for a long time^(17)^.

#### Criteria for admission

If the child has severe acute malnutrition^(18)^: It is diagnosed by weight-for-height below −3 sd of the WHO standards, by MUAC <11⋅5 cm, and by clinical signs like bilateral oedema^(19)^.

#### Kwashiorkor or oedematous malnutrition

It is also a form of severe undernutrition, the child's muscles were wasted, but wasting may not be apparent due to generalised oedema or swelling because of excess fluid in the tissues^(19)^.

#### Criteria for discharging children from treatment

Weight-for-height/length is ≥ –2 *Z*-scores and having no oedema for at least 2 weeks, or MUAC is ≥125 mm and no oedema for at least 2 weeks^(19)^.

#### Household food insecurity access scale

Household food insecurity access scale (HFIAS) gave the highest prevalence estimates of food insecurity, as might be expected given its inclusion of less severe manifestations of insecurity, including psychological anxiety and food consumption preferences^(20)^.

### Data processing and analysis

Data were implicated, entered into Ep-data version 4.2 software, and transferred to SPSS for windows version 25 software for analysis. The occurrence of missing values, possible outliers and multicollinearity was checked by exploratory analysis. The wealth index was calculated using principal component analysis (PCA), based on PCA expectations. Accordingly, the households were considered into three wealth terciles for further analysis.

**Fig. 1.**
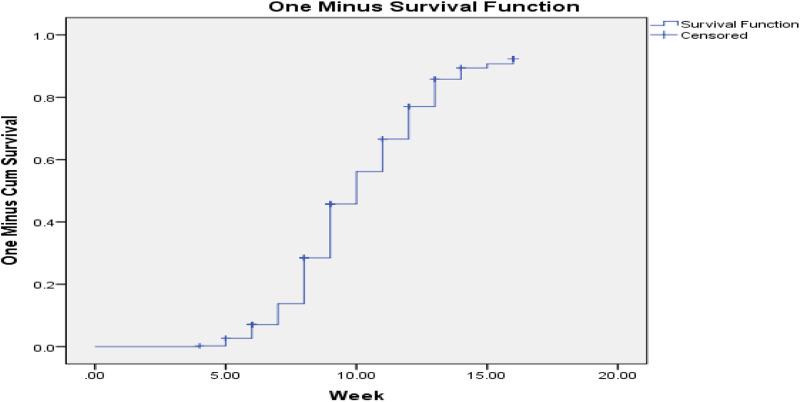
One minus survival function test for severe acute malnourished children cured for MUAC for overall children in West Hararghe Zone, Oromia, Ethiopia.

**Fig. 2.**
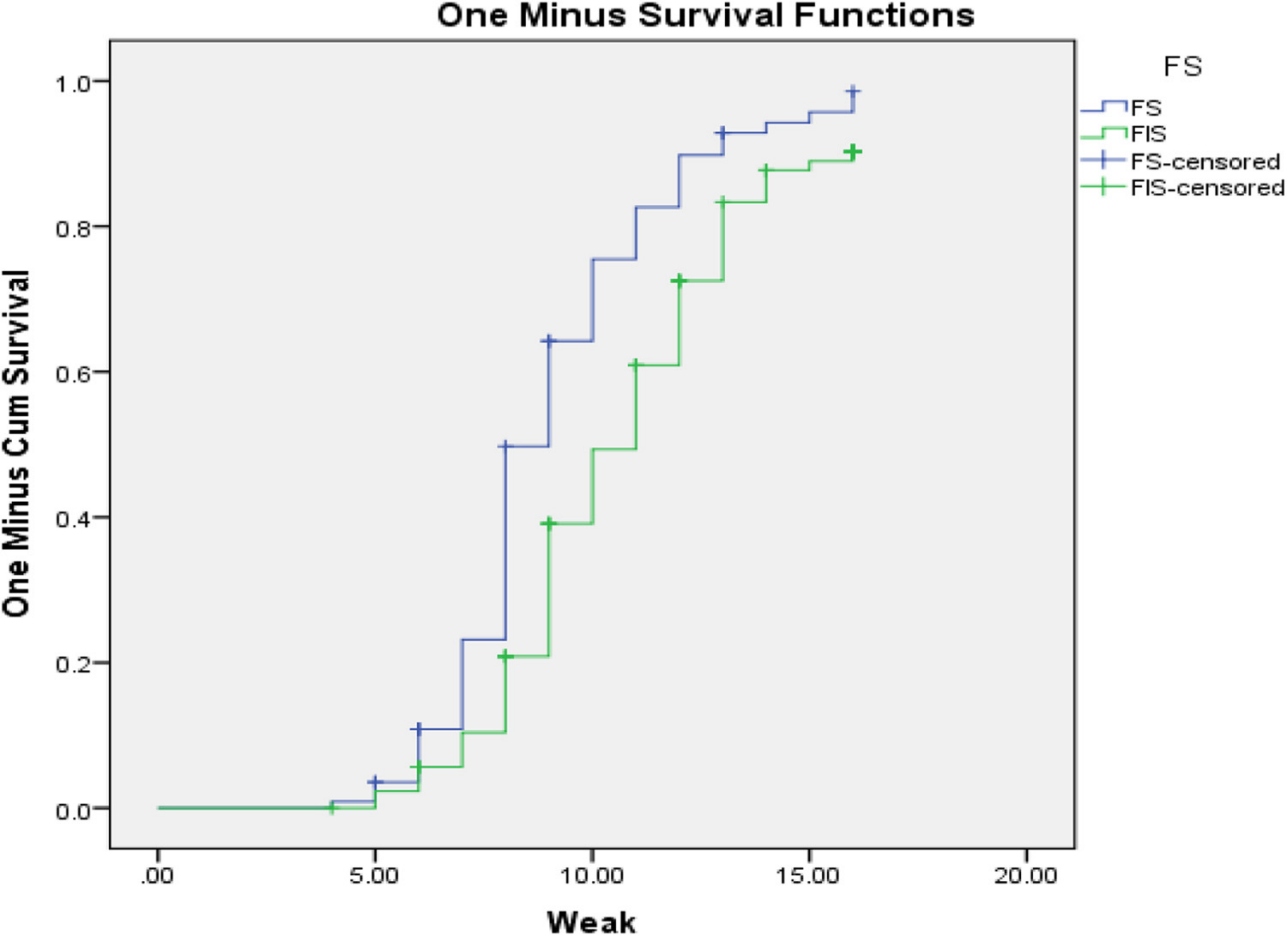
One minus survival function test for severe acute malnourished for SAM children cured for MUAC from food insecure households in West Hararghe Zone, Oromia, Ethiopia.

**Fig. 3.**
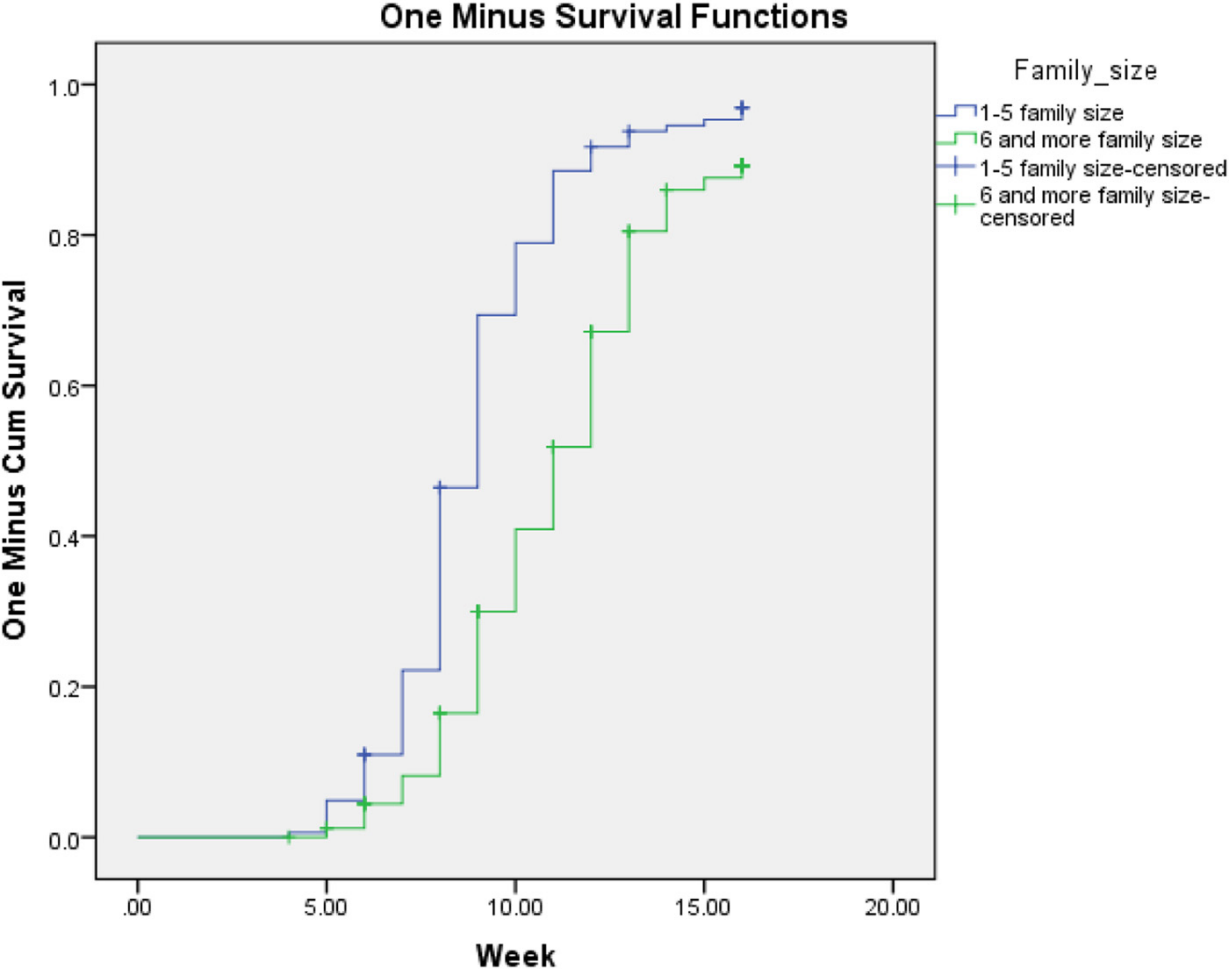
One minus survival function test for severe acute malnourished children cured for MUAC from household members seven and more family households in West Hararghe Zone, Oromia, Ethiopia.

Both bi-variate and multivariable Cox regression analyses were done. Kaplan Meier hazard curve with the log-rank test was fitted to identify the occurrence of a difference in recovery rate among the categorical variables. Mantel-cox and generalised Wilcoxon tests of equality of survival distributions are significant and one minus survival function line is parallel for those candidate variables of multivariable Cox regression (Figs 1–3).

For the different levels, under-five children with SAM cases followed in weeks from admission to the occurrence of the event (cured). Person time was calculated and the incidence was determined. In the present study, person time was reported in child week. Child week is the total follow-up times of each child from admission to the occurrence of the events (cured or censored).

Those variables with *P* ≤ 0⋅25 in the bi-variate Cox regression were selected for the multivariable Cox regression analysis. All statistical tests were measured significantly at *P*-values of < 0⋅05.

## Result

### Socio-economic characteristics

In the present study, 414 under-five children's participated of them 257 (62 %) were females and the reaming were males. When we come to the age of the majority of under-five children were below 24 months 311 (75⋅1 %), on the other hand, more than half of the children were from the household with family members of six and above 250 (60⋅4 %), the rest were below six family members. When we come to the household wealth index from the participants 166 (40⋅1 %) households were under the lowest tercile of the rank in a similar way majority of households or family members of under-five children admitted with severe acute malnutrition were food insecure with 302 (72⋅9 %) (Table 1).

**Table 1.** Socio-demographic characteristics of under-five children and their family in Habro Worada, West Hararghe Zone, Oromia Region, Ethiopia

Variable	Frequency%
Sex	
Male	157 (37⋅9)
Female	257 (62⋅1)
Age in months	
6–11	137 (33⋅1)
12–23	174 (42⋅0)
24–35	56 (13⋅5)
36–47	37 (8⋅9)
48–60	10 (2⋅4)
Family size in household	
<6 members	164 (39⋅6)
6 and above members	250 (60⋅4)
Household wealth index	
Low	166 (40⋅1)
Medium	172 (41⋅5)
High	76 (18⋅4)
Household food security status	
Food secure	112 (27⋅1)
Food insecure	302 (72⋅9)

### Treatment outcome

**Fig. 4.**
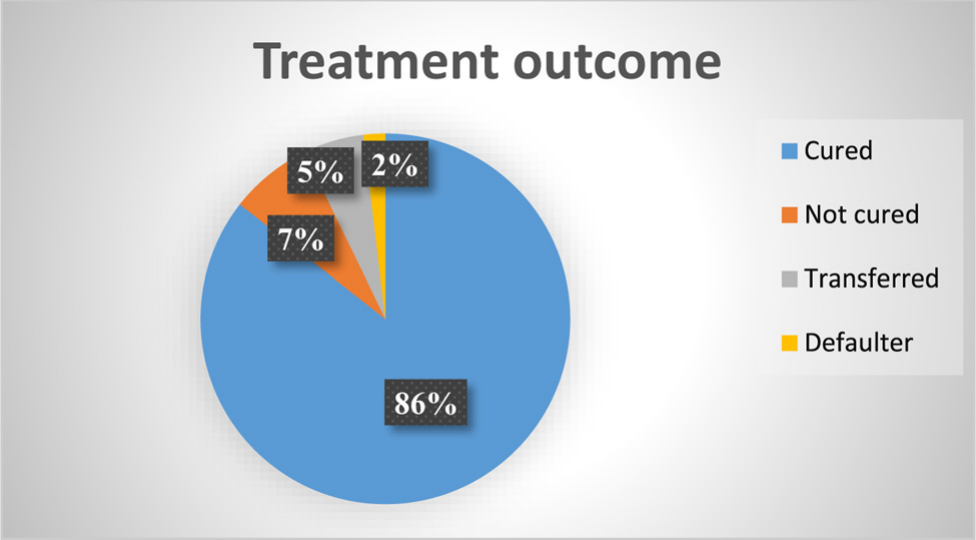
Treatment outcome of severe acute malnourished children for MUAC in health post in Habro Woreda, West Hararghe Zone, Oromia Region, Ethiopia.

In the present study, children were followed for 16 weeks after admission for severe acute malnutrition in health posts. From the total follow-up, 354 (85⋅5 %) were cured during treatment and the remaining were not cured, transferred to the stabilisation centre and defaulters of the treatment were 30 (7⋅2 %), 22 (5⋅3 %) and 8 (1⋅9 %), respectively (Fig. 4).

### Time in the week for survival from severe acute malnutrition by MUAC

In the present study, the minimum week for the cure was 4 weeks, the maximum was 16 weeks and the overall time survival from severe acute malnutrition by MUAC is determined as 10 weeks with 10 weeks, 95 % CI (9⋅65–10⋅35). However, time to cure varies with different variables, for example; at the age of 6–11 months cure time becomes 11 weeks becomes 95 % CI (10⋅47–11⋅53) and at the age of 12–23 months, it becomes 9 weeks becomes 95 % CI (8⋅66–9⋅34). In addition, family members with six and more members have a median time for MUAC is 11 weeks with 95 % CI (10⋅51–11⋅49). In the same way, among food insecure food-insecure 11 weeks with 95 % CI (10⋅48–11⋅52), among the lowest wealth index 11 weeks with 95 % CI (10⋅41–11⋅59), and with the highest wealth index the cure time will fall to 9 weeks with 95 % CI (8⋅23–9⋅77) (Table 2).

**Table 2.** Medians week for survival from severe acute malnutrition by MUAC in Habro Woreda, West Hararghe Zone, Oromia Region, Ethiopia

Variable	Median estimate week	95 % confidence Interval
Overall children	10	9⋅65, 10⋅35
Sex		
Male	10	9⋅31, 10⋅69
Female	10	9⋅56, 10⋅44
Age of children in months		
6–11	11	10⋅47, 11⋅53
12–23	9	8⋅66, 9⋅34
24–35	10	9⋅38, 10⋅62
36–47	10	7⋅76, 12⋅24
48–60	10	9⋅65, 10⋅35
Family size		
1–5 family size	9⋅000	8⋅70, 9⋅30
6 and more family size	11⋅000	10⋅51, 11⋅49
Household food security		
Food secured	9⋅000	8⋅55, 9⋅45
Food insecured	11⋅000	10⋅48, 11⋅52
Wealth index category		
Lowest	11	10⋅41, 11⋅59
Medium	10	9⋅44, 10⋅56
High	9	8⋅23, 9⋅77

MUAC, mid-upper arm circumference.

### Factors associated with survival status of children for MUAC

Factors associated with cure time to MUAC in Proportional Hazards Model, after adjusting for related variables to control confounders and testing for Kaplan–Meier test to identify parallel line assumptions. Under-five children with severe acute malnutrition from family size 6 and above member were 2⋅16 times at higher risk for long duration in treatment (AHR = 2⋅1, 95 % CI 1⋅53–3⋅06) compared to lower family size. Children from low wealth index category family members have a 1⋅4 times hazard of long-term treatment for severe acute malnutrition to cure for MUAC (AHR = 1⋅40, 95 % CI: 1⋅095–1⋅770). In addition, SAM children from food-insecure households were 2⋅61 times at higher risk during treatment by MUAC compared with food secured households (AHR = 2⋅61, 95 % CI 1⋅53, 3⋅06) (Table 3).

**Table 3.** Multivariable Cox proportional hazards model identifying the determinants of time to cure among children with severe acute malnutrition (18) for MUAC in Habro Worada, West, Hararghe Zone, Oromia Region, Ethiopia

Variable	*Β*	*P*-value	AHR	95 % CI
Sex				
Male	−	−	1	−
Female	−0⋅009	0⋅935	0⋅99	0⋅79, 1⋅24
Age in months				
6–11	0⋅42	0⋅480	1⋅52	0⋅48, 4⋅83
12–23	0⋅83	0⋅156	2⋅30	0⋅73, 7⋅26
24–35	0⋅85	0⋅156	2⋅35	0⋅72, 7⋅64
36–47	0⋅42	0⋅490	1⋅53	0⋅46, 5⋅06
48–60	−	−	1	−
Family size in household				
<6 members	−	−	1	−
6 and above members	0⋅75	0⋅001	2⋅16	1⋅53, 3⋅06*
Wealth index				
Low	0⋅33	0⋅007	1⋅40	1⋅095, 1⋅770*
Medium	−0⋅15	0⋅497	0⋅86	0⋅559, 1⋅325
High	−	−		−
Household food security				
Food secure	−	−	−	−
Food insecure	0⋅77	0⋅001	2⋅16	1⋅53, 3⋅06*
Admission MUAC	0⋅23	0⋅68	1⋅259	0⋅98, 1⋅60

MUAC, mid-upper arm circumference; AHR, adjusted hazard ratio; CI, confidence interval.

## Discussion

In the present study, the minimum and maximum time to cure is determined as 4 and 16 weeks, respectively, and the time to cure severe acute malnutrition by MUAC is determined as 10 weeks. The following factors associated with time to cure were family size, wealth index and household food insecurity associated with cure time by MUAC from severe acute malnutrition.

A study conducted in Dire Dawa among different health institutions suggests the median recovery time was 8⋅7 weeks^(21)^ and another study from North Gondar Zone, reveals that the median time to recovery was 38⋅5 days or 5 weeks^(22)^. However, in the present study, the median week for survival was determined as 10 weeks for MUAC and this is a higher time to stay in treatment. This may be because the above study durations were based on target weight gain from admission but the discharge criteria in this study were on target MUAC or 12⋅5 cm. This may suggest us children who are declared cured and discharged from severe acute malnutrition management programme by weight gain were may not cure of MUAC.

When we come to the factors associated with time to cure; in the present study, family size is associated with time to cure. The present study is similar to the retrospective study conducted among under-five children admitted to the therapeutic feeding unit at Dubti referral hospital^(23)^. This may be because children in a large family may face several problems that may include lack of care, competition for supplementary treatment food, Plumpy'nut or ‘Nefis Aden’, among severely malnourished children and other family members.

Another factor associated with time is the wealth index in a parallel way study conducted at Dubti referral hospital; Afar region reveals that household income in Ethiopian Bir (ETB) is associated with time to cure^(23)^. Even though there are different ways that means study conducted in Afar is followed by target weight as discharge criteria but in the present study, we have followed MUAC <12⋅5 cm as discharge criteria for severely malnourished children. The similarity of the cure time among poor or low wealth index households in different studies may low income or being poor directly affect cure time.

On another hand in the present study household food insecurity, one of the associated factors with time to cure this may be because of food-insecure children from the food insecure household food-insecure with severe acute malnutrition more and it may result in long cure time from the SAM^(24)^.

### Practical implication

MUAC bids an advantage on screening malnutrition and following up the progress of the intervention on malnutrition particularly in developing countries and hard to reach area MUAC is easy to care out. In addition to that MUAC is easy for utilisation and there is no need of age of a child, which minimises relaying on weight and age. Cure by MUAC is longer than that of weight and we have suggested that discharge of MUAC less than 12⋅5 cm were result in relapse or repeated admission^(25)^.

## Conclusion

In the present study, the median time to cure severe acute malnutrition by MUAC is determined as 10 weeks. Moreover, family size 6 and above members, low wealth index category and household food insecurity were identified as risks to stay at treatment for MUAC.

### Recommendation

From the present study, we recommend that it is better to use MUAC as diagnosing and discharge criteria for severe acute malnutrition among under-five children and better to increase treatment week to 10 and above or until MUAC becomes 12⋅5 cm for all under-five children irrespective of admission criteria.
